# Serum amlyoid A: An inflammatory adipokine mediating postburn outcomes

**DOI:** 10.1002/ctm2.412

**Published:** 2021-06-06

**Authors:** Abdikarim Abdullahi, Mile Stanojcic, Nancy Yu, Osai Samadi, Ali‐reza Sadri, Roohi Vinaik, Natalie Coburn, Marc G. Jeschke

**Affiliations:** ^1^ Faculty of Medicine University of Toronto Toronto Ontario Canada; ^2^ Biological Sciences Sunnybrook Research Institute Toronto Ontario Canada; ^3^ Odette Cancer Center Sunnybrook Hospital Toronto Ontario Canada; ^4^ Ross Tilley Burn Centre Sunnybrook Hospital Toronto Ontario Canada; ^5^ Department of Surgery Division of Plastic Surgery University of Toronto Toronto Ontario Canada; ^6^ Department of Immunology University of Toronto Toronto Ontario Canada

Dear Editor,

Burn trauma is associated with increased mortality rates despite many key advances and therapeutic strategies to improve patient care.^1^ One of the main drivers of poor outcomes in these patients is the complex yet overwhelming chronic inflammation that occurs in response to the injury.^2^ Unfortunately, the mechanisms, and in particular the signaling circuitry that facilitates persistent inflammation in these patients are essentially unknown. We report here that the acute phase protein serum amyloid A (SAA) mediates chronic inflammation and poor outcomes in both burn and septic burn patients. The inflammatory and prognostic potential of SAA has only been previously studied in context of heart disease and neonatal sepsis, leaving its potential pathogenic role in other conditions largely unexplored.^3^, ^4^ Therefore, in this present study we first characterized the kinetics of circulating SAA in both burn patients and septic burn patients relative to nonburn controls (Supplemental Table 1). Interestingly, circulating SAA levels persisted post the acute phase (>14 days) in both patient groups, with septic burn patients showing a greater increase in magnitude at both time (acute and late) points relative to burn patients (Figure 1A and B). Furthermore, when we profiled SAA levels in burn patients who succumbed to the injury, SAA levels were significantly higher in the nonsurvivors relative to burn patients who survived the injury (Figure 1C). Increased plasma SAA levels also paralleled an increase in the pro‐inflammatory cytokine interleukin 1 beta (IL‐1β), with septic burn patients showing a greater increase at both time (acute and late) points relative to nonseptic burn patients (Figure 1D). We further explored the inflammatory inducing effects of SAA using differentiated human THP‐1 monocytes (Figure 1E). Differentiated human THP‐1 macrophages stimulated with recombinant human SAA showed dramatic increases in the gene and protein expression of pro‐inflammatory cytokines IL‐6 and IL‐1β (Figure 1F–I). Interestingly, SAA stimulation in macrophages evoked a similar pro‐inflammatory cytokine profile to that of lipopolysaccharide (LPS), a well‐established pro‐inflammatory endotoxin. In corroboration of our clinical findings, mice subjected to either a 30% total body surface area burn injury and or a two‐hit burn + *Pseudomonas aeruginosa* (PA) infection to mimic postburn sepsis, and also showed a greater increase in plasma SAA levels and IL‐1β, with burn septic mice showing a greater increase in magnitude relative to the burn group (Figure S1A–C). To more directly link chronic SAA levels with poor outcomes, we injected septic burn mice with recombinant SAA daily for 1 week. Daily administration of SAA in septic burn mice significantly induced lethality and increased mortality (100 vs. 20%) relative to untreated septic burn mice (Figure S1D). Additionally, bone marrow–derived macrophages (BMDMs) isolated from postburn mice stimulated with SAA also showed dramatic increases in the gene and protein expression of the pro‐inflammatory cytokines IL‐6 and IL‐1β (Figure S2A–E).

**FIGURE 1:**
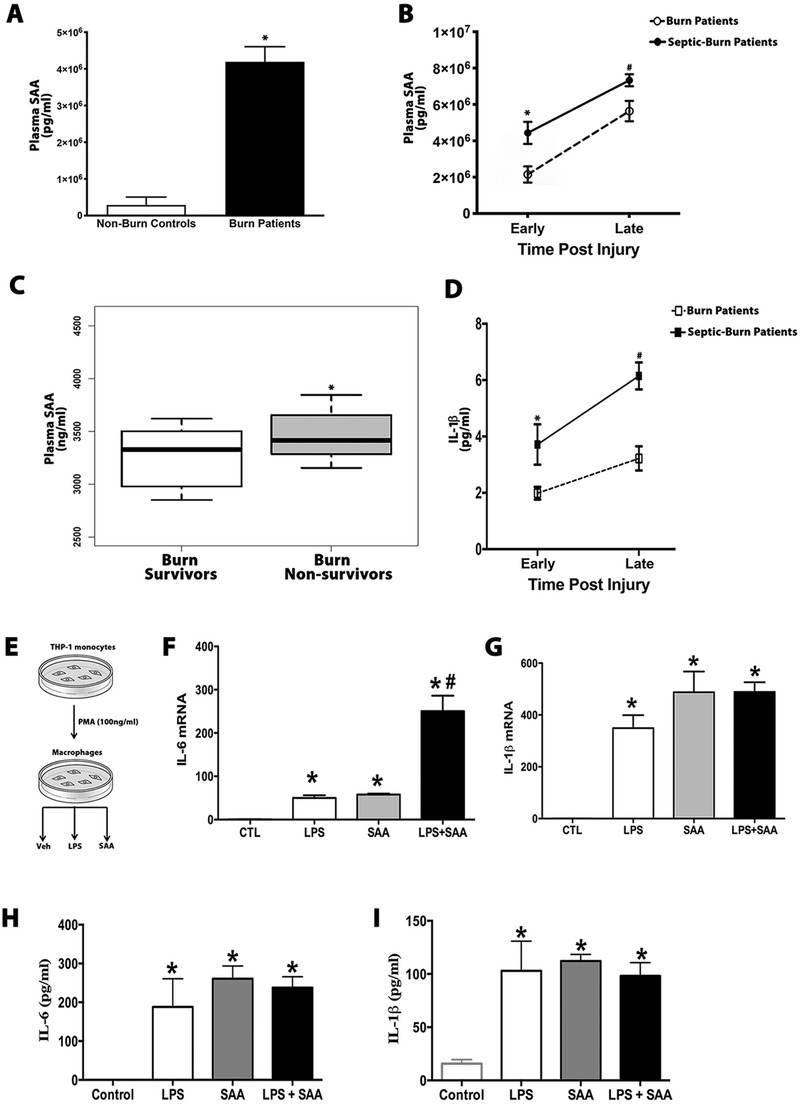
Serum amyloid A (SAA), a potent pro‐inflammatory inducer in macrophages, is persistently elevated in burn and septic burn patients. (A) SAA levels were measured in plasma of nonburn controls and burn patients during the acute phase (24 h postinjury). (B) Circulating SAA levels were measured in plasma from nonburn controls, burn patients, and septic burn patients at early (0–3 days) and late (>14 days) time points postinjury. (C) Plasma SAA levels from burn patients who either survived or succumbed to the burn injury. (D) Circulating IL‐1β levels were measured in plasma from burn patients and septic burn patients at early (0–3 days) and late (>7days) time points postinjury. (E) Schematic illustration of human THP‐1 monocytes differentiated to macrophages via PMA incubation (100 ng/ml) for 48 h and then treated accordingly. (F–G) Quantitative RT‐PCR analysis of pro‐inflammatory genes IL‐6 and IL‐1β measured in THP‐1 differentiated macrophages treated with either vehicle, lipopolysaccharide (LPS; 100 ng/ml), and or recombinant human SAA (0.5 μg/ml) for 24 h. (H–I) Circulating proinflammatory cytokines IL‐6 and IL‐1β measured in culture medium obtained from THP‐1 differentiated to macrophages treated with either vehicle, LPS (100 ng/ml), and or recombinant human SAA (0.5 μg/ml) for 24 h. Data represented as mean ± SEM, *p* < 0.05 * = significant difference versus nonburn controls, * = significant difference versus early time burn patients, * = significant difference versus burn survivors. # = significant difference versus late time point burn patients (*N* = 10–20). Data represented as mean ± SEM, *p* < 0.05 * = significant difference versus control, # = significant difference versus SAA or LPS (*N* = 10)

Although the liver has previously been reported to be the main production site for SAA, recent findings in obese patients have implicated the adipose tissue as another key producer of SAA.^5^, ^6^, ^7^, ^8^ Given the metabolic etiology of obese patients (insulin resistance, hyperglycemia, inflammation) also experienced by burn patients following the injury, we hypothesized that the chronic circulating SAA levels observed in burn patients may arise from the adipose tissue. To test this hypothesis, we measured SAA expression in subcutaneous white adipose tissue (sWAT) collected from burn patients and nonburn controls (Table S1). sWAT from burn patients showed significant gene and protein expression of SAA compared to nonburn controls (Figure 2A and B). These findings were further corroborated by our ex vivo fat culture findings, in which secretomes from cultured sWAT isolated from burn patients showed increased SAA release compared to sWAT isolated from nonburn controls (Figure 2C). Interestingly, SAA protein expression in the sWAT of postburn patients was a late response, in which elevated expressions occurred after the acute phase (>7days) postinjury (Figure 2D).

**FIGURE 2:**
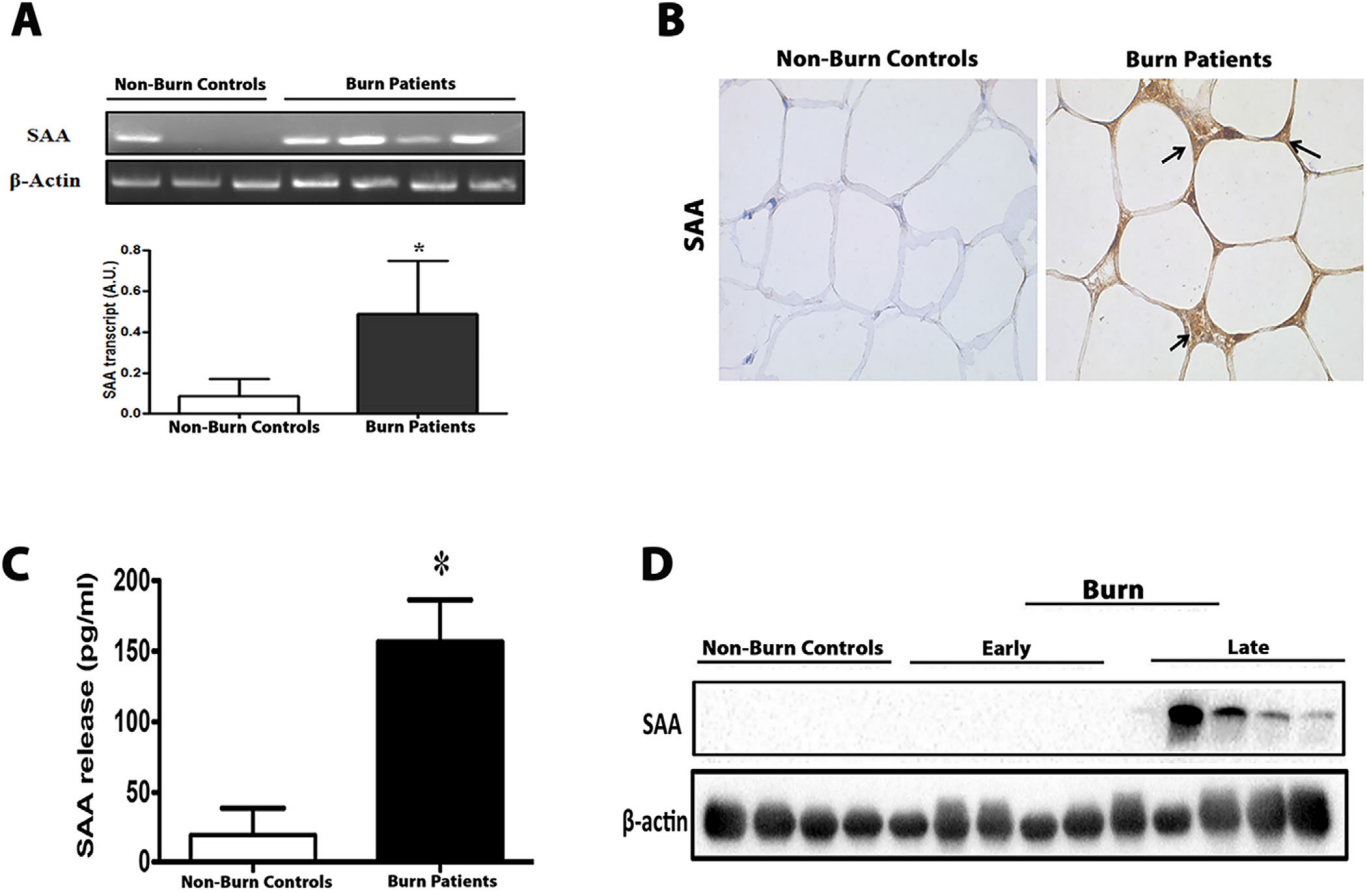
The adipose tissue is a source of serum amyloid A (SAA) release after burn trauma. (A) Adipose SAA mRNA levels measured by quantitative real‐time RT‐PCR in subcutaneous white adipose tissue isolated from nonburn controls and burn patients 7 days postinjury. (B) Histological staining of SAA protein in subcutaneous white adipose tissue obtained from nonburn controls and burn patients 7 days postinjury. (C) Circulating SAA protein in culture medium obtained from ex vivo fat explants isolated from nonburn controls and burn patients 7 days postinjury. (D) Western blot protein expression of SAA in subcutaneous white adipose tissue obtained from nonburn controls and burn patients during early (3 days) and late (14 days) time points postinjury. Data represented as mean ± SEM, *p* < 0.05 * = significant difference versus nonburn controls (*n* = 8–10)

It has been reported that the production of SAA is controlled by a variety of cytokines released during inflammatory states that include IL‐6 and IL‐1β.^6^ Cytokine profiling of both burn patients and postburn mice revealed significant elevations of plasma IL‐6 levels relative to nonburn controls and remained elevated over the course of the injury (Figure 3A–D). To determine the functional role of IL‐6 in postburn SAA production, we used IL‐6 knock‐out (KO) mice (Figure 3E). Consistent with a role in the regulation of SAA production, IL‐6^−/−^ mice showed no elevations in SAA production compared to WT mice postburn injury (Figure 3F). To rule out IL‐1β as another potential regulator of postburn SAA production, we also assessed SAA production in postburn and septic burn mice using NLRP3 KO mice (NLRP3^−/−^) that are impaired in IL‐1β production. In contrast to IL‐6^−/−^ mice, NLRP3^−/−^ mice subjected to either a burn injury alone or burn + PA infection showed no impairments in SAA production, suggesting that IL‐1β is expendable for SAA production after a burn injury (Figure 3A and B). Furthermore, studies outsides of sepsis and burns have implicated macrophages as both producers and effector cells of SAA. To test this hypothesis, we pharmacologically depleted macrophages using clodronate‐containing liposomes (Figure 3G and H). Surprisingly, postburn mice depleted of macrophages showed no significant differences in hepatic SAA production relative to postburn mice (Figure 3I and J), suggesting that macrophages are also expendable for the postburn SAA production and surge.

**FIGURE 3:**
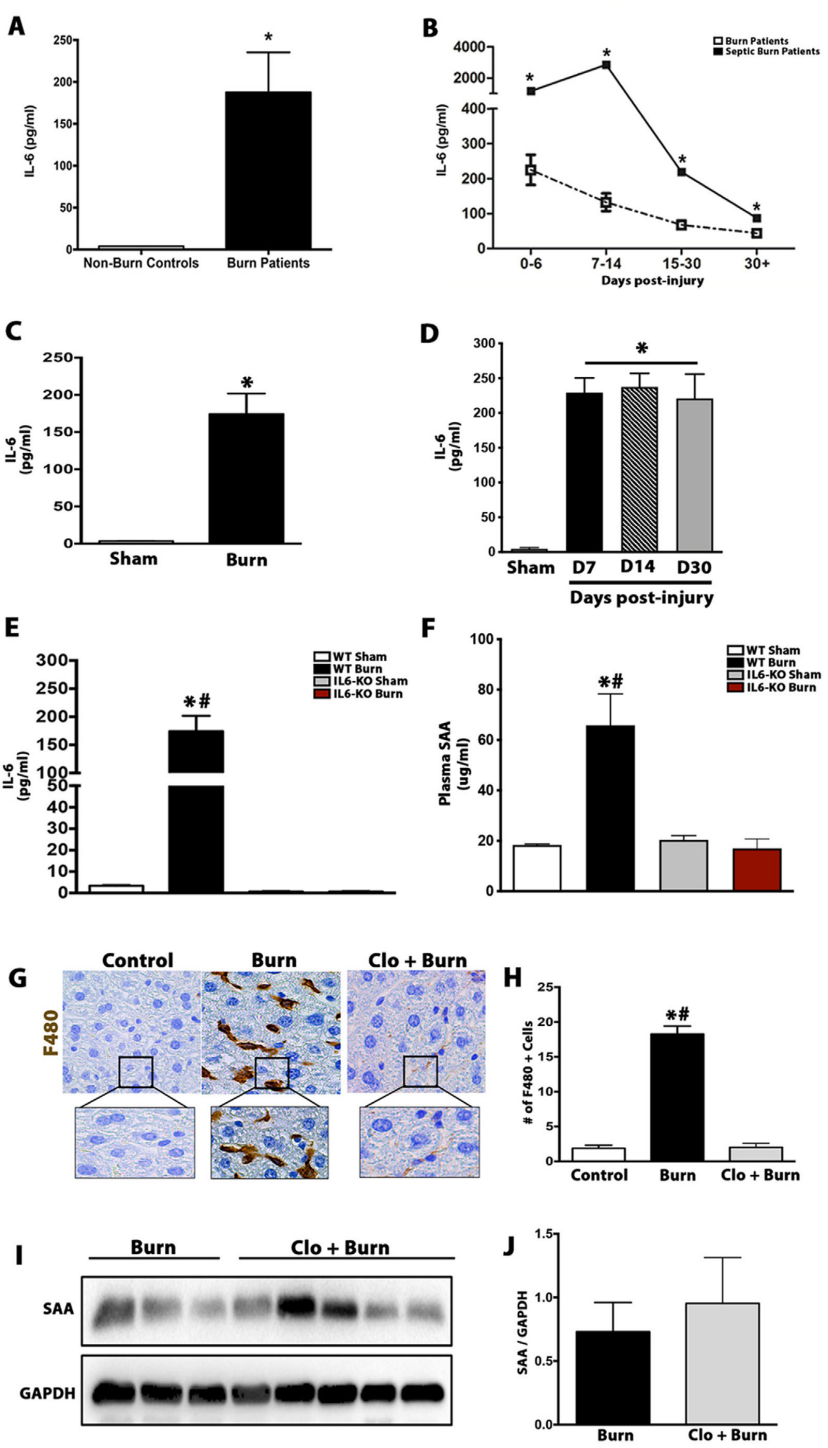
IL‐6 is chronically elevated in both burn patients and post‐burn mice and is required for burn‐induced SAA production. (A) IL‐6 levels measured in plasma obtained from burn patients and nonburn controls 24 h postinjury. (B) IL‐6 levels measured in plasma from burn patients and septic burn patients over the course of the injury. (C) IL‐6 levels measured in plasma from postburn mice and littermate controls 24 h postinjury. (D) IL‐6 levels measured in plasma from postburn mice and littermate controls over time. (E) IL‐6 levels measured in plasma obtained from sham, WT + burn, IL‐6KO sham, and IL‐6KO+burn 24 h postinjury. (F) Circulating SAA levels measured in plasma obtained from sham, WT + burn, IL‐6KO sham, and IL‐6KO + burn 24 h postinjury. (G–H) Representative histological staining and quantification of F480 in liver sections from WT mice treated with vehicle or clodronate‐containing liposomes postburn injury. (I–J) Immunoblot analysis and densitometry quantification of SAA protein in WT mice treated with vehicle or clodronate‐containing liposomes postburn injury. Data represented as mean ± SEM, *p* < 0.05 * = significant difference versus non‐burn controls, *p* < 0.05 * = significant difference versus burn patients (*N* = 20). Data represented as mean ± SEM, *p* < 0.05 * = significant difference versus sham mice. *p* < 0.05 # = significant difference versus WT + burn, (*n* = 8). *p* < 0.05 * = significant difference versus controls. *p* < 0.05 # = significant difference versus Clo + burn, (*n* = 10)

We next investigated the therapeutic potential of attenuating SAA levels postburn injury through pharmacological inhibition of its main regulator IL‐6 through the drug Tocilizumab.^9^, ^10^ Treatment with the IL‐6R blocker for 1‐week in postburn mice not only significantly reduced plasma SAA levels but was also not associated with toxicity‐induced mortality (Figure 4A and B). Administration of IL‐6R blocker in burned mice significantly decreased both hepatic and adipose SAA expression compared to nontreated mice and was associated with decreased circulating levels of IL‐1β (Figure 4C–E). In conclusion, our findings not only highlight SAA as a key player in postburn outcomes but that SAA may also serve as a valuable prognostic marker of burn‐associated sepsis that can be targeted therapeutically to improve outcomes.

## CONFLICT OF INTEREST

The authors declare that they have no conflicts of interest.

## Supporting information

Supplemental Figure 1: Chronic persistent SAA levels in burn and septic burn mice. (A) SAA levels were measured in the plasma of sham and postburn mice 24 h postinjury. (B) Circulating SAA levels were measured in the plasma of sham, burn, and burn + PA mice 7 days postinjury. (C) Circulating proinflammatory cytokine IL‐1β measured in the plasma of sham, burn, and burn + PA mice 7 days postinjury. (D) Kaplan–Meier survival curve of burn + PA and burn + PA mice injected with one dose of recombinant human SAA (2 mg/kg) postinjury. Data are represented as mean ± SEM, *p* < 0.05* = significant difference versus sham; *p* < 0.05* = significant difference versus burn + PA; *p* < 0.05# = significant difference versus burn (*N* = 6–8).
Supplemental Figure 2: SAA induces a pro‐inflammatory response in bone marrow–derived macrophages isolated from postburn mice. (A) Schematic illustration of the bone marrow‐derived macrophages isolated from postburn mice and treatment experiment. (B‐C) Quantitative RT‐PCR analysis of pro‐inflammatory genes IL‐6 and IL‐1β measured in bone marrow‐derived macrophages (BMDMs) isolated from postburn mice treated with either vehicle, LPS (100 ng/ml), and or recombinant human SAA (0.5 μg/ml) for 24 h in culture medium. (D–E) Circulating IL‐6 and IL‐1β levels in culture medium obtained from bone marrow‐derived macrophages (BMDMs) treated with either vehicle, LPS (100 ng/ml), and or recombinant human SAA (0.5 μg/ml) for 24 h in culture medium. Data represented as mean ± SEM, *p* < 0.05 * = significant difference versus controls (*N* = 10).
Supplementary Figure 3: NLRP3 is dispensable in septic and burn‐induced SAA production. (A) Plasma SAA levels measured over time in NLRP3 KO mice subjected to both a burn injury. (B) Plasma SAA levels measured in NLRP3 KO mice subjected to both a burn injury and PA infection. Data represented as mean ± SEM, *p* < 0.05 * = significant difference versus sham, (*n* = 5).
Supplementary Figure 4: Blockade of IL‐6 signaling attenuates burn‐induced SAA and IL‐1β secretion. (A) Kaplan–Meier survival curves of mice injected postburn injury either with vehicle or IL‐6R blocker (1 mg/day) for 1 week. (B) SAA levels were measured in the plasma of sham, burn, and IL‐6R blocker burn treated mice 1‐week posttreatment. (C) SAA mRNA expression measured in the livers of sham, burn, and IL‐6R blocker burn treated mice 1‐week posttreatment. (D) Histological staining of SAA in subcutaneous adipose tissue of sham, burn, and IL‐6R blocker burn treated mice 1‐week posttreatment. (E) Circulating proinflammatory cytokine IL‐1β was measured in plasma of sham, burn, and IL‐6R blocker burn treated mice 1‐week posttreatment. Data represented as mean ± SEM, *p* < 0.05 * = significant difference versus sham, # = significant difference versus Burn + IL‐6R, (*n* = 6).
Supplementary Figure 5: Schematic illustration serum amyloid A in the pathogenesis of burn of injury. The image illustrates the pathogenic role of SAA in mediating postburn inflammation and mortality as well as the adipose tissue serving as a source of SAA production post the acute phase to burn injury.
