# Supplementary material for: Serum amlyoid A: An inflammatory adipokine mediating postburn outcomes
Source: Clin Transl Med. 2021 Jun 6;11(6):e412. doi: 10.1002/ctm2.412 (PMC8181199; doi:10.1002/ctm2.412)
Supplement: Supplementary file 1 — Supplemental Figure 1: Chronic persistent SAA levels in burn and septic burn mice. (A) SAA levels were measured in the plasma of sham and postburn mice 24 h postinjury. (B) Circulating SAA levels were measured in the plasma of sham, burn, and burn + PA mice 7 days postinjury. (C) Circulating proinflammatory cytokine IL‐1β measured in the plasma of sham, burn, and burn + PA mice 7 days postinjury. (D) Kaplan–Meier survival curve of burn + PA and burn + PA mice injected with one dose of recombinant human SAA (2 mg/kg) postinjury. Data are represented as mean ± SEM, p < 0.05* = significant difference versus sham; p < 0.05* = significant difference versus burn + PA; p < 0.05# = significant difference versus burn (N = 6–8). Supplemental Figure 2: SAA induces a pro‐inflammatory response in bone marrow–derived macrophages isolated from postburn mice. (A) Schematic illustration of the bone marrow‐derived macrophages isolated from postburn mice and treatment experiment. (B‐C) Quantitative RT‐PCR analysis of pro‐inflammatory genes IL‐6 and IL‐1β measured in bone marrow‐derived macrophages (BMDMs) isolated from postburn mice treated with either vehicle, LPS (100 ng/ml), and or recombinant human SAA (0.5 μg/ml) for 24 h in culture medium. (D–E) Circulating IL‐6 and IL‐1β levels in culture medium obtained from bone marrow‐derived macrophages (BMDMs) treated with either vehicle, LPS (100 ng/ml), and or recombinant human SAA (0.5 μg/ml) for 24 h in culture medium. Data represented as mean ± SEM, p < 0.05 * = significant difference versus controls (N = 10). Supplementary Figure 3: NLRP3 is dispensable in septic and burn‐induced SAA production. (A) Plasma SAA levels measured over time in NLRP3 KO mice subjected to both a burn injury. (B) Plasma SAA levels measured in NLRP3 KO mice subjected to both a burn injury and PA infection. Data represented as mean ± SEM, p < 0.05 * = significant difference versus sham, (n = 5). Supplementary Figure 4: Blockade of IL‐6 signaling attenuates bur [file CTM2-11-e412-s001.pdf]

Supplemental Table 1: Clinical Demographics

	Non-Burn Controls (n=8)	Burn (n=36)	Burn Sepsis (n=16)
**Age (days)**			
Mean ±SD	46±7	44.6±11	52.7±13
**Gender**			
Male	5 (63%)	31 (86%)	12 (75%)
Female	-	5 (14%)	4 (25%)
**Survival Status**			
Survivor	100%	31 (86%)	10 (63%)
Non-survivor	-	5 (14%)	6 (37%)
**Burn Etiology**			
Flame	-	30 (83%)	16 (100%)
Scald	-	5 (14%)	0 (0%)
Electrical	-	1 (3%)	0 (0%)

Supplemental Figure 1

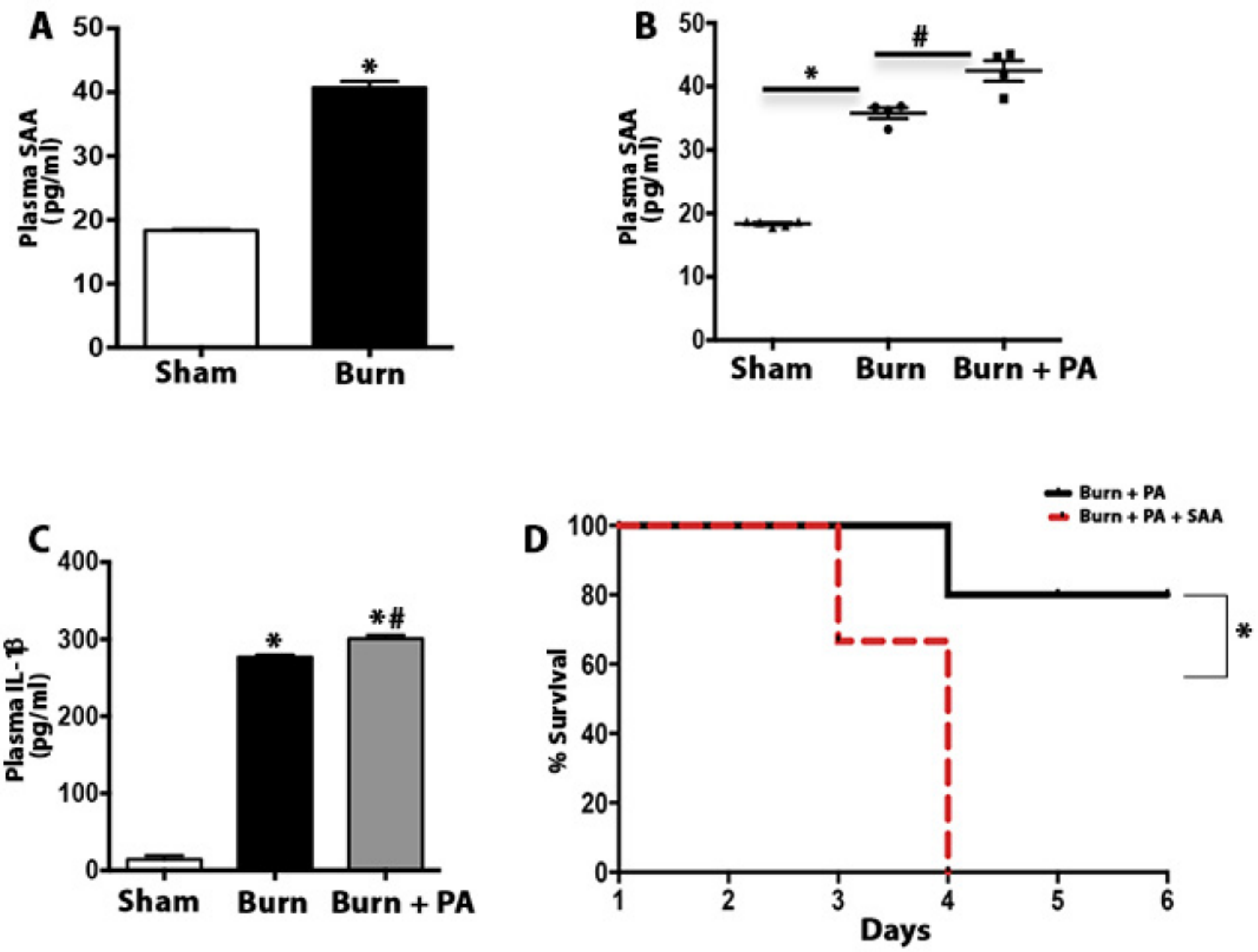

Supplemental Figure 2

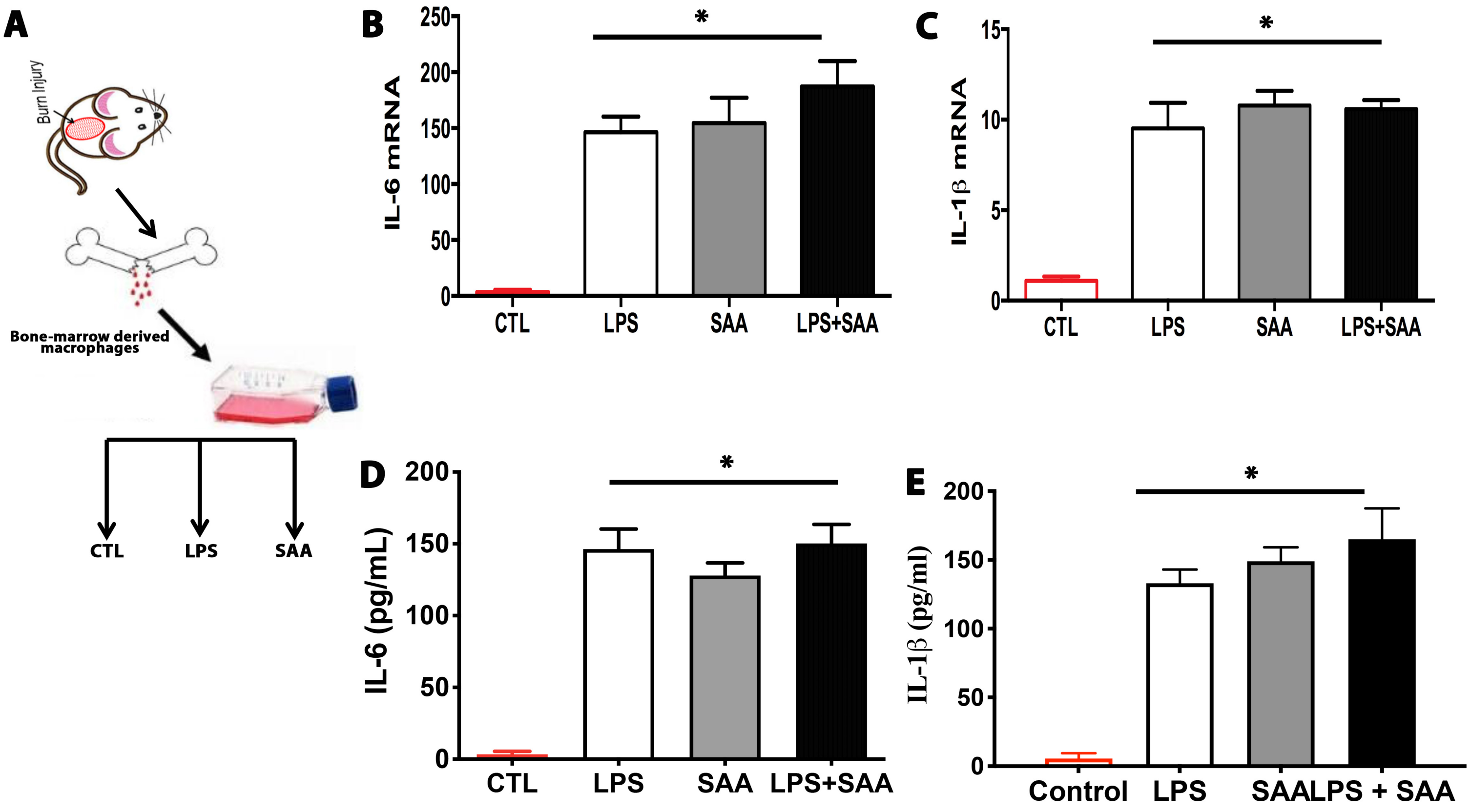

Supplemental Figure 3

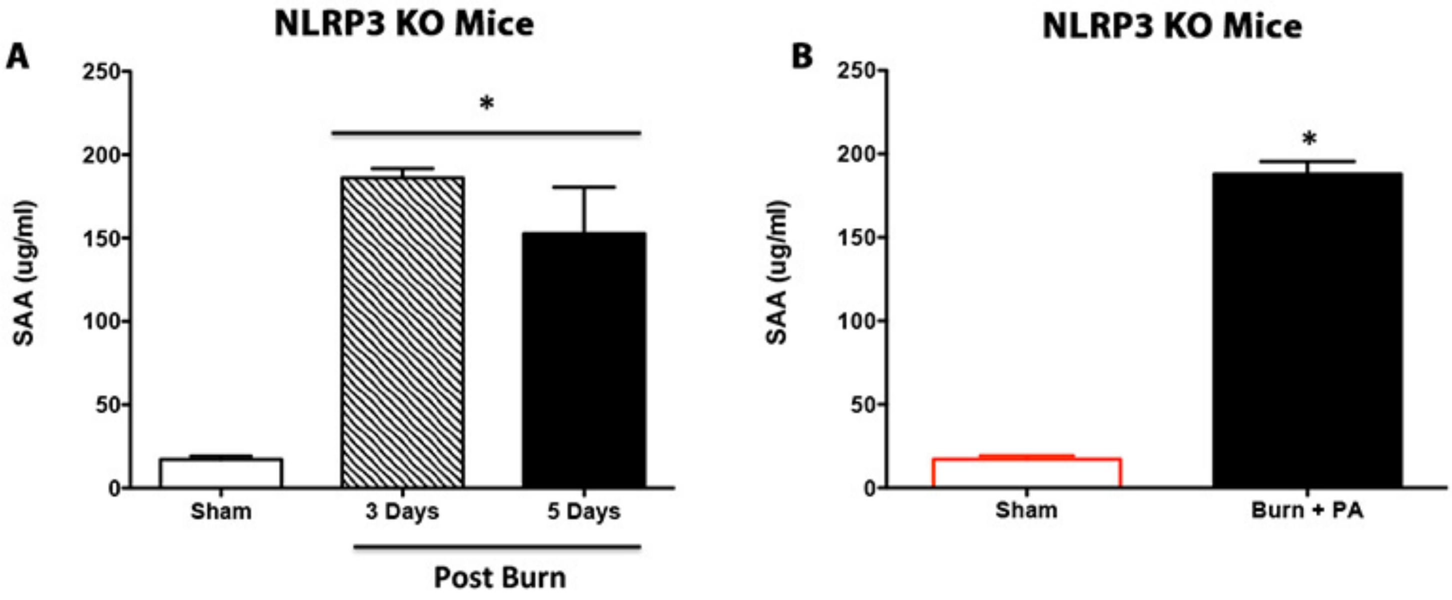

Supplemental Figure 4

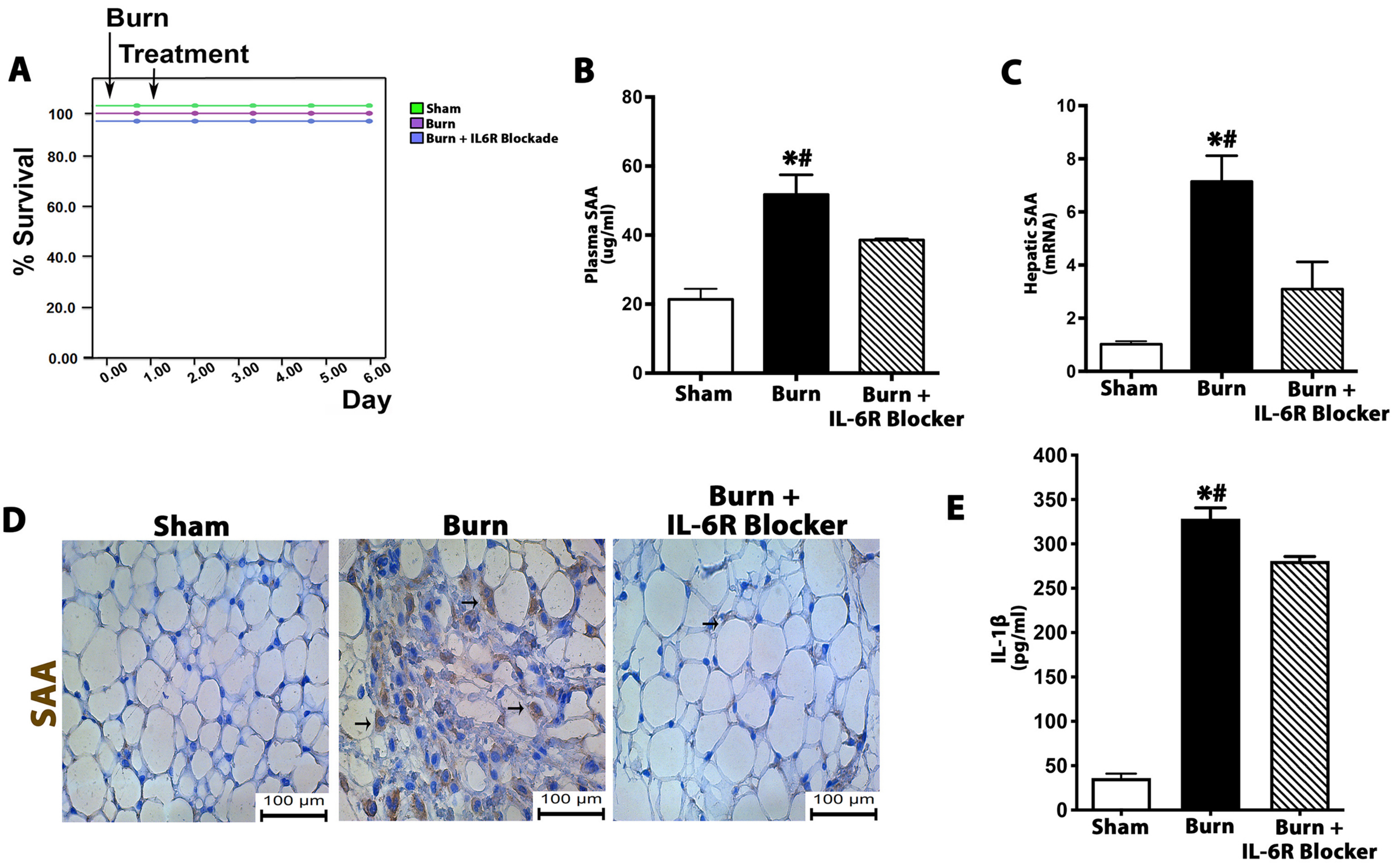

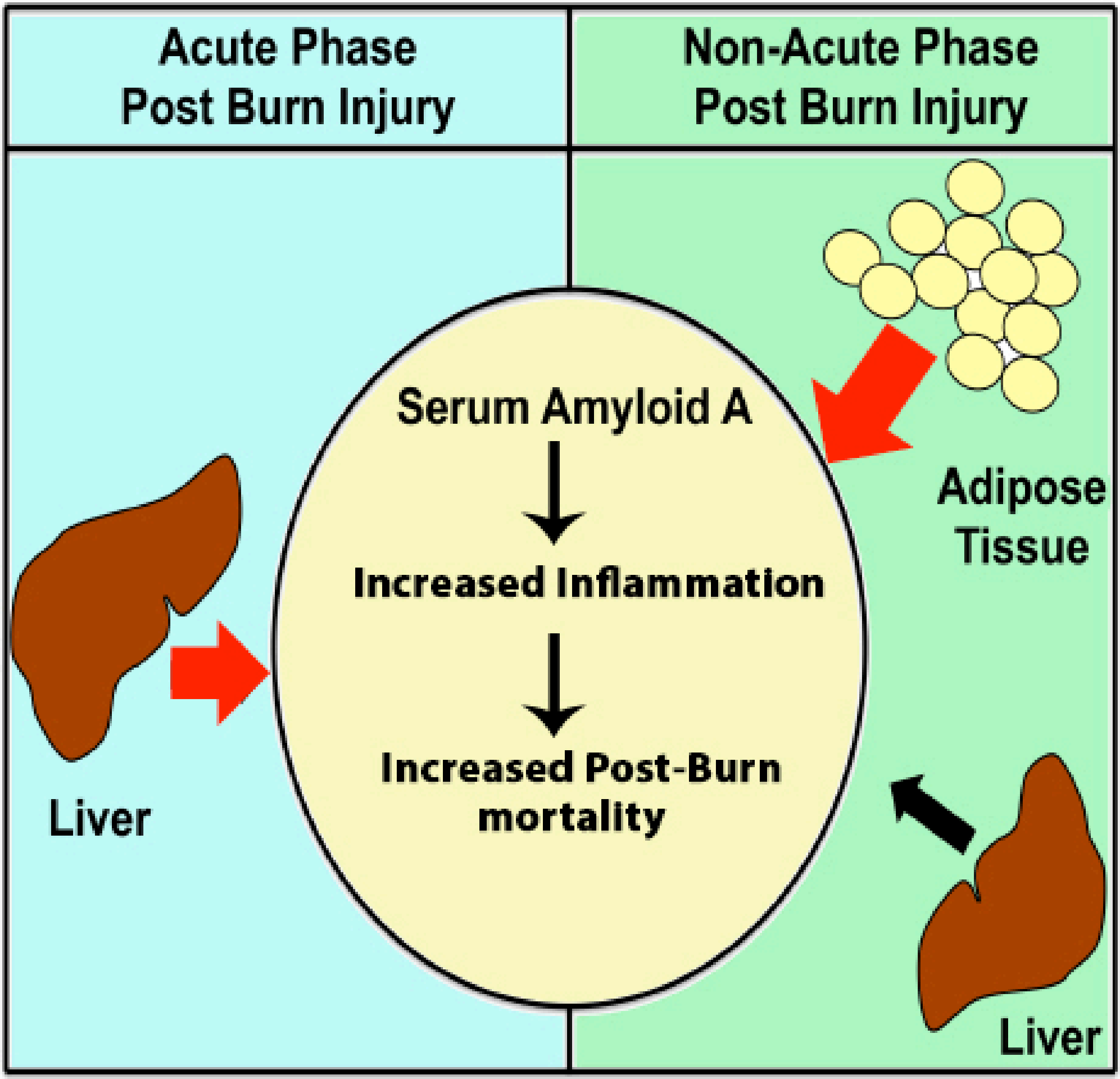

Supplemental Figure 5
